# Dynamic and Assembly of Benthic Bacterial Community in an Industrial-Scale In-Pond Raceway Recirculating Culture System

**DOI:** 10.3389/fmicb.2021.797817

**Published:** 2021-12-23

**Authors:** Yiran Hou, Bing Li, Gangchun Xu, Da Li, Chengfeng Zhang, Rui Jia, Quanjie Li, Jian Zhu

**Affiliations:** ^1^Key Laboratory of Freshwater Fisheries and Germplasm Resources Utilization, Ministry of Agriculture and Rural Affairs, Freshwater Fisheries Research Center, Chinese Academy of Fishery Sciences, Wuxi, China; ^2^Wuxi Fisheries College, Nanjing Agricultural University, Wuxi, China; ^3^Ocean and Fishery Research Institute of Rizhao, Rizhao, China

**Keywords:** in-pond raceway recirculating culture systems, co-occurrence network, bacterial communities, purification area, community assembly mechanisms, stochastic processes

## Abstract

To reduce water utilization, limit environmental pollution, and guarantee aquatic production and quality, the in-pond raceway recirculating culture system (IPRS) has been developed and is widely used. The effectiveness and sustainability of IPRSs rely on a good understanding of the ecological processes related to bacterial communities in the purification area. In this study, we investigated the dynamics and assembly mechanisms of benthic bacterial communities in the purification area of an industrial-scale IRPS. We found significant temporal and spatial variations in the sediment characteristics and benthic bacterial communities of the IPRS, although correlation analyses revealed a very limited relationship between them. Among the different culture stages, we identified numerous benthic bacteria with different abundances. Abundances of the phyla Bacteroidota and Desulfobacterota decreased whereas those of Myxococcota and Gemmatimonadota increased as the culture cycle progressed. Co-occurrence networks revealed that the bacterial community was less complex but more stable in the IPRS at the final stage compared with the initial stage. The neutral community model (NCM) showed that stochastic processes were the dominant ecological processes shaping the assembly of the benthic bacterial community. The null model suggested that homogenizing dispersal was more powerful than dispersal limitation and drift in regulating the assembly of the community. These findings indicate that the benthic microbial communities in purification areas of the IPRS may not be affected by the deposited wastes, and a more stable benthic microbial communities were formed and mainly driven by stochastic processes. However, the benthic microbial communities in the purification area at the end of the culturing stage was characterized by potentially inhibited organic matter degradation and carbon and sulfur cycling abilities, which was not corresponding to the purification area’s function. From this point on, the IPRS, especially the purification area was needed to be further optimized and improved.

## Introduction

The Food and Agriculture Organization of the United Nations predicted that the world’s food supply will need to increase by 70% to meet the demand of the rapidly growing population until 2050 (FAO, 2016). Aquatic products represent one of the most important food sources, and the aquaculture industry has expeditiously expanded (Wang et al., 2015). China is the most prosperous aquaculture country, producing > 70% of the aquatic products that are used worldwide (Mo et al., 2017). Aquaculture pond, as a typically aquatic ecosystem with artificial nutrient input, has become increasingly important (FAO, 2019).

Traditional pond aquaculture relies on increasing the breeding density in order to increase the production and economic benefits. However, increased breeding density requires the addition of great quantities of feed into the ponds during the aquaculture cycle (Gao et al., 2019). Most of these substances cannot be absorbed by the cultured species and are deposited at the bottom of the culture ponds (Nhan et al., 2006). Long-term accumulation of these nutrient-rich materials in sediments can destroy the ecological balance of the aquaculture system, leading to the death of cultured animals through eutrophication (Thurlow et al., 2019; Liu et al., 2020).

To solve these issues, recirculating aquaculture systems (RASs) have been developed as eco-responsible alternatives to traditional aquaculture systems to improve the efficiency and sustainability of aquaculture (Badiola et al., 2018; Xiao et al., 2019). Especially, the in-pond raceway recirculating culture system (IPRS) is a better type of RAS which have been applied in freshwater aquaculture successfully (Martins et al., 2010; Wang et al., 2019). The IPRS is a closed pond aquaculture system with separated intensive culture and purification areas (Li et al., 2019; Wang et al., 2019). The intensive culture area accounts for 2–5% of the total pond area and consists of several aquaculture raceways (Li et al., 2019; Wang et al., 2019). The water is re-used after mechanical and biological treatment to reduce the consumption of water and promote resource utilization efficiency in the culture ponds (Zhang et al., 2011; Li et al., 2019). The IPRS was first introduced to China by the United States Soybean Export Council in 2011. Since then, IPRSs were implemented in more than 18 provinces and cities after several upgrades and improvements (Li et al., 2019; Wang et al., 2019). Studies of the IPRS are of great significance for improving aquaculture methods to achieve optimal balance between increasing production and ecological sustainability.

The composition of bacterial communities in the aquaculture environment can serve as an indicator to reflect the ecological status of the ecosystem (Zhao et al., 2020). Excessive nutrients in the aquaculture environment can change the dynamics of bacterial communities, which are closely related to the health of breeding species and are also affected by external environmental factors (Xue et al., 2017). In contrast, some bacteria in aquaculture environments can accelerate the decomposition of residual feed and feces to purify the water (Tan et al., 2016). Previous studies have described the bacterial communities in RASs, especially in biological filters where many bacteria accumulate during culture activities (Ruan et al., 2015; Huang et al., 2016; Ma et al., 2021).

The community assembly and the mechanisms that shape bacterial community diversity are important but poorly understood topics in aquatic ecosystems. From the meta-community perspective, the bacterial community assembly is influenced by both selective and non-selective processes (Chen et al., 2019). They include deterministic abiotic (environmental factors such as temperature and salinity) and biotic factors (species interactions such as predation and competition) (Chave, 2004) as well as stochastic processes, such as birth, death, immigration, limited dispersal, and drift (Vanwonterghem et al., 2014). Several researchers evaluated the dynamics of bacterial communities in RASs with different scales and rearing species and reported that environmental factors have obvious effects on bacterial communities (Huang et al., 2020; Bao et al., 2021; Ma et al., 2021). However, little is known about the assembly of bacterial communities in IPRSs during the aquaculture process.

In this study, we used an industrial scale IPRS established in Yancheng, Jiangsu Province, China (Supplementary Figure 1). We collected sediments from different locations in the purification area of the IPRS at the initial, middle, and final stages of the culture cycle. We assessed sediment characteristics and evaluated the benthic bacterial communities using high-throughput sequencing of the 16S rRNA gene and then compared the data among the sampling periods. A co-occurrence network was used to evaluate the changes in community complexity and stability over the culture cycle. The mechanisms that shaped benthic bacterial communities in the IPRS were analyzed using the neutral community model (NCM) and null model. We also assessed whether the shaping of benthic bacterial communities in the purification area of the IPRS was governed more by deterministic processes mediated by excessive nutrients in the system or by stochastic processes.

## Materials and Methods

### Sample Collection and DNA Extraction

This study was conducted at Jianhu Guoneng New Energy Development Co., Ltd., Jiangsu Province, China (N 119°46′12.52″, E 33°20′10.60″). The studied IPRS system covered an area of about 306,700 square meters and consisted of two separate intensive culture areas and a purification area (Supplementary Figure 1). There were 20 and 30 aquaculture raceways (22 × 5 × 2.5 m) constructed in the two separate intensive culture areas, respectively. The raceway consisted of an air lifting and water pushing device, a fish culture area, bottom rechargeable aerobic facilities, and a waste collecting area (Supplementary Figure 2). The air lifting and water pushing device constantly pushed the water from the aquaculture raceway into the purification area. The waste collecting area collected the aquaculture wastes, such as food, feces, and other organic detritus, carried in the water. However, the collection efficiency of the waste collection area was < 30%, so most of the aquaculture waste flowed into the purification area, where it settled (Yu and Wang, 2016).

At the beginning of the culture activities, 12,000 grass carp (*Ctenopharyngodon idella*) seedlings (average 10 g per individual) were added to each of the aquaculture raceways. The feeding trial lasted for 4 months, and the fish were fed a commercial diet (4% weight of the individuals) three times a day. After the culture activities, the average survival rate of grass carps was 90.12 ± 0.05%, which met the expectations of normal culturing production. Eight sampling locations (stations O1–O8) were set up at equal distances along the water recirculating path in the purification area, from which surface sediments (0–5 cm thick) were sampled on June 27th (Initial), August 29th (Middle), and October 29th (Final) of 2020. For each sample, about 50 g of surface sediment were collected, temporarily held on ice, and transported to the laboratory within 6 h. A 10 g aliquot of sediment from each sample was immediately preserved in liquid nitrogen and stored at –80°C until DNA extraction. The remaining sediments were dried in a Martin Christ Gefriertrocknungsanlagen Alpha 1–4 LD plus lyophilizer (Harz, Germany) for 48 h at –50°C, ground to powder in a mortar to pass through a 100 μm mesh size sieve, and stored in a freezer at −20°C for analysis of sediment characteristics. DNA was extracted from samples using the FastDNA Spin Kit for Soil (MP Biomedicals, Irvine, CA, United States). The concentration and integrity of extracted DNA were determined using a NanoDrop device (Thermo Fisher Scientific, Carlsbad, CA, United States) and 2% agarose gel electrophoresis, respectively.

### Analysis of Sediment Characteristics

The total organic carbon (TOC) and total nitrogen (TN) concentrations in sediments were measured using a FlashEA 1112 Series NC Analyzer. Before the analysis, the homogenized and freeze-dried sediment samples were acidified with 6 mol/L HCl at 25°C for 24 h to remove inorganic carbon. The acidified sediment samples then were oven-dried to a constant weight for 48 h at 50°C. The total protein (TP) concentration in the sediment was determined following the SMT protocol (Ruban et al., 1999; Ruban and Rauret, 2001).

### 16S rRNA Gene Amplification and Sequencing

The V3–V4 regions of 16S rRNA genes from each extracted DNA sample were amplified using primers 341F–806R (341F: ACTCCTACGGGAGGCAGCAG; 806R: GGACTACHVGG GTWTCTAAT) (Berg et al., 2012). All PCRs were performed in 15 μL reaction volumes containing 7.5 μL of Phusion^®^ High-Fidelity PCR Master Mix (New England Biolabs, Ipswich, MA, United States), 1 μL of forward and reverse primers (10 μM), 1 μL of dNTPs (2.5 mM), and 1 μL template DNA. Thermal cycling consisted of initial denaturation at 95°C for 2 min followed by 25 cycles of denaturation at 94°C for 5 s, annealing at 55°C for 30 s, and elongation at 72°C for 30 s. The PCR products from each sample were detected by electrophoresis on 1.5% agarose gel. The obtained PCR products were purified, quantified, and mixed in equal amounts to construct the sequencing libraries. The library quality was evaluated using an Agilent Bioanalyzer 2100 system and a Qubit @2.0 Fluorometer (Thermo Fisher Scientific). Finally, the Illumina NovaSeq 6000 platform (San Diego, CA, United States) was applied to sequence these libraries using the 250 bp paired-end strategy.

### Data Processing

Reads with an average Phred score (Q score) < 20 and those containing ambiguous bases, homopolymers > 6, mismatches in primers, and sequence length < 150 bp were deleted from the datasets (Bokulich et al., 2013). Remaining high-quality reads were assigned to the samples based on their unique barcodes at the end of the reverse primers. Subsequently, reads with overlap longer than 10 bp and without any mismatch were assembled into tags using FLASH (Magoc and Salzberg, 2011). Tags with ≥ 97% similarity were assigned to the same operational taxonomic unit (OTU) using the QIIME v1.9.2 (Caporaso et al., 2010) software package. The representative sequence of each OTU was chosen by the default method and assigned a bacterial taxon based on the SILVA database (Yilmaz et al., 2014). A bacterial OTU abundance table was constructed and normalized using a standard number of tags according to the sample with the least number of tags.

### Statistical Analysis

Alpha diversity indices, including Chao1 and Shannon indices, were calculated using the “vegan” package in R v4.0.2 for each of the benthic bacterial communities. The differences in sediment characteristics and bacterial alpha diversity indices among samples from different culture stages and different locations at the same stage were analyzed using Tukey’s honest significant difference (HSD) test. Variations in the benthic bacterial community compositions of samples from different culture stages and different locations at the same stage were evaluated using Principal Coordinate Analysis (PCoA) and the PERMANOVA test based on the Bray-Curtis distance in the “vegan” package in R.

A stacked bar graph was used to show the relative abundance of dominant bacterial phyla in sediments of the IPRS. Boxplots were used to illustrate the distribution of benthic bacteria at the phylum level, and differences at different culture stages were also evaluated using Tukey’s HSD test. The co-occurrence networks of benthic bacterial communities in different culture stages were constructed based on Spearman rank correlations among the OTUs that occurred in at least 60% of samples. Co-occurrence events were identified based on statistically robust correlations (| correlation coefficient| > 0.8 with *p*-value < 0.05) (Junker and Schreiber, 2008). The *p-*values were adjusted using the Benjamini-Hochberg method, and the obtained networks were visualized in Gephi v0.9.1 (Bastian et al., 2009).

### Mechanisms Involved in Regulating Benthic Bacterial Community Structure

To determine the potential importance of stochastic processes in determining benthic bacterial community structure, we used an NCM to predict the relationship between OTU detection frequency and the relative abundance of OTUs in collected samples (Sloan et al., 2016). In this model, *Nm* is an estimate of dispersal between communities. The parameter *Nm* determines the correlation between occurrence frequency and regional relative abundance, with N describing the community size and m being the immigration rate. The parameter R2 represents the overall fit to the neutral model. The 95% confidence intervals around all fitting statistics were calculated by bootstrapping with 1,000 bootstrap replicates.

Null model analysis also was performed to calculate the relative contribution of five ecological processes (homogeneous selection, heterogeneous selection, homogenizing dispersal, dispersal limitation, and drift) to shaping the bacterial community assembly (Stegen et al., 2013). The processes governing benthic bacterial community structure were identified based on the β-nearest taxon index (βNTI) and the Raup-Crick metric (RC). The fraction of pairwise comparisons with βNTI < −2 was considered to be the percentage of homogeneous selection, whereas that with βNTI > 2 was considered to be the percentage of heterogeneous selection. Next, the taxonomic diversity metric, RC, was used to partition the remaining pairwise comparisons with |βNTI| ≤ 2. The fraction of pairwise comparisons with RC < −0.95 was treated as the percentage of homogenizing dispersal, whereas the fraction with RC > 0.95 was treated as the dispersal limitation. The remaining comparisons with |βNTI| ≤ 2 and |RC| ≤ 0.95 represented the percentage of drift.

## Results

### Temporal and Spatial Variations of Sediment Characteristics

**FIGURE 1:**
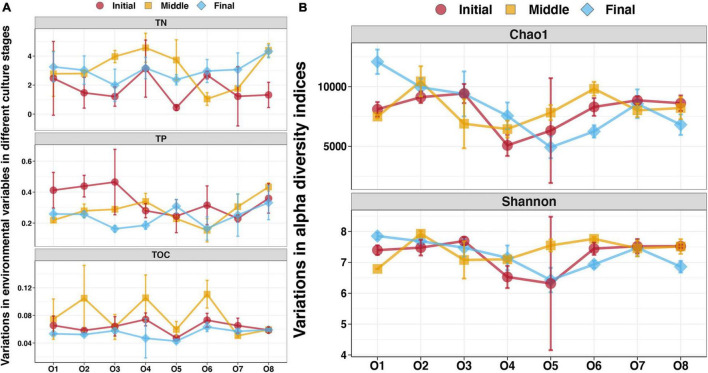
**(A)** Sediment characteristics at different locations in the purification area of the IPRS during the culture cycle. **(B)** Alpha diversity indices of the benthic bacterial communities at different locations in the purification area of the IPRS during the culture cycle.

To evaluate the enrichment of sedimentary nutrients in the purification area of the IPRS, the distribution of TN, TP, and TOC in the sediments at different locations during the culture cycle was investigated (Figure 1A). Significantly higher TN contents were found in the sediments at the initial stage compared with those from the middle and final stages (Tukey’s HSD test, *p* < 0.05, Supplementary Table 1). The concentrations of TP in the sediments were highest in the initial stage, second highest in the middle stage, and lowest in the final stage, and the difference between the initial and final stages was statistically significant (Tukey’s HSD test, *p* < 0.05, Supplementary Table 1). The TOC contents were significantly higher in the sediments at the middle stage compared with the initial and final stages (Tukey’s HSD test, *p* < 0.05, Supplementary Table 1).

For sediments from different locations in the same season, TP and TN in the initial stage, TOC in the middle stage, and all three of them in the final stage were stable throughout the purification area of the IPRS (Tukey’s HSD test, *p* > 0.05, Supplementary Table 2). The TOC contents of sediments at the initial stage were highest at sampling station O4 and lowest at O5, and the values differed significantly (Tukey’s HSD test, *p* < 0.05, Supplementary Table 2). The concentrations of TN and TP in sediments at the middle stage also differed among stations, with higher values at stations O4 and O8 and lower values at O1 and O6 (Tukey’s HSD test, *p* < 0.05, Supplementary Table 2).

### Temporal and Spatial Variations of Benthic Bacterial Diversity

We detected 72 benthic bacterial communities from eight locations in the IPRS purification area during the culture cycle using Illumina sequencing technology based on the bacterial 16S rRNA gene. Overall, 3,320,634 tags (average of 46,120 tags per sample, ranging from 30,019 to 59,857) were obtained. After quality control, these tags were clustered into 27,642 OTUs for taxonomic annotation of the benthic bacterial communities. The Chao1 and Shannon diversity indices were used to calculate diversity of the benthic bacterial communities (Figure 1B). No significant variation of these two indices was found in samples from the purification area of the IPRS during the culture cycle (Tukey’s HSD test, *p* > 0.05, Supplementary Table 3). Values of the Chao1 and Shannon indices were also consistent among samples from different locations at the initial stage (Tukey’s HSD test, *p* > 0.05, Supplementary Table 4). At the middle stage, however, values of both indices were significantly higher at station O2 and O6 than at the other stations (Tukey’s HSD test, *p* < 0.05, Supplementary Table 4). These two stations were located in the direct drainage area of the breeding areas. The Chao1 and Shannon indices of the benthic bacterial communities were highest and lowest at stations O1 and O5, respectively, at the final stage of the culture cycle (Figure 1B). When we assessed the relationship between benthic bacterial diversity and sediment characteristics in the purification area of the IPRS, no significant correlations were found among stations at a given time point or at a given station over time (Spearman correlation, *p* > 0.05).

### Temporal and Spatial Variations of Benthic Bacterial Communities

**FIGURE 2:**
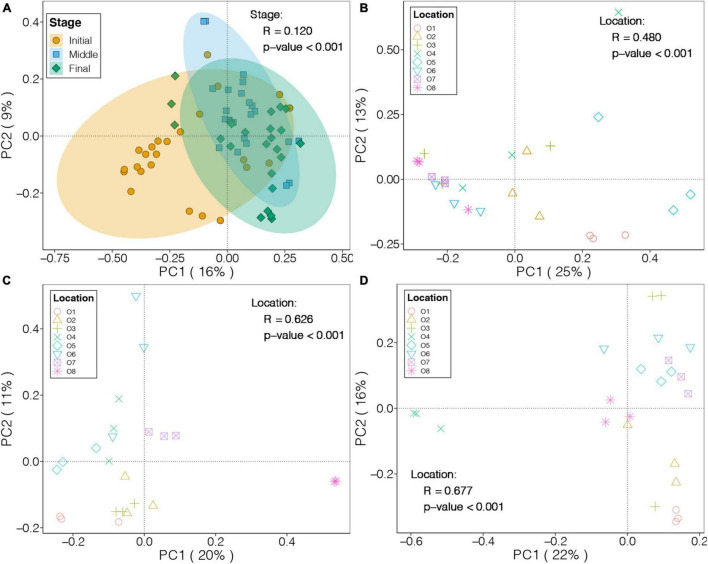
PCoA and PERMANOVA results for the benthic bacterial communities in the purification area of the IPRS during **(A)** the culture cycle and **(B–D)** each culture stage (**B**, initial; **C**, middle; and **D**, final).

We applied PCoA based on Bray-Curtis distance to investigate the differences in the benthic bacterial communities in the purification area of the IPRS at different culture stages (Figure 2A). The first two PCs explained 16 and 9% of the total variation in the benthic bacterial communities, respectively, and samples from the different stages were separately clustered. PERMANOVA also demonstrated that culture stage had significant effects on the benthic bacterial communities in the purification area of the IPRS (*p* < 0.05), as it explained 12% of the total variation.

We also performed PCoA on the benthic bacterial communities at each culture stage, and the first two PCs explained 38, 31, and 38% of the total variation in the initial, middle, and final stages, respectively (Figures 2B–D). Moreover, the location of sediments in the purification area of the IPRS had a significant influence on the benthic bacterial communities at all three culture stages (PERMANOVA, *p* < 0.05), as it explained 48, 63, and 68% of the total variation in the benthic bacterial communities in the initial, middle, and final stages, respectively.

The Mantel test was used to explore potential relationships between the composition of benthic bacterial communities and sediment characteristics. No significant correlation was found between TN, TP, and TOC content of the sediments and the benthic bacterial communities (Mantel test, *p* > 0.05). Within a given culture stage, only TOC content was significantly correlated with the benthic bacterial communities at the final stage (Mantel test, *p* < 0.05, Supplementary Table 5).

### Variations of Benthic Bacterial Taxa and Co-occurrence Patterns During the Culture Cycle

**FIGURE 3:**
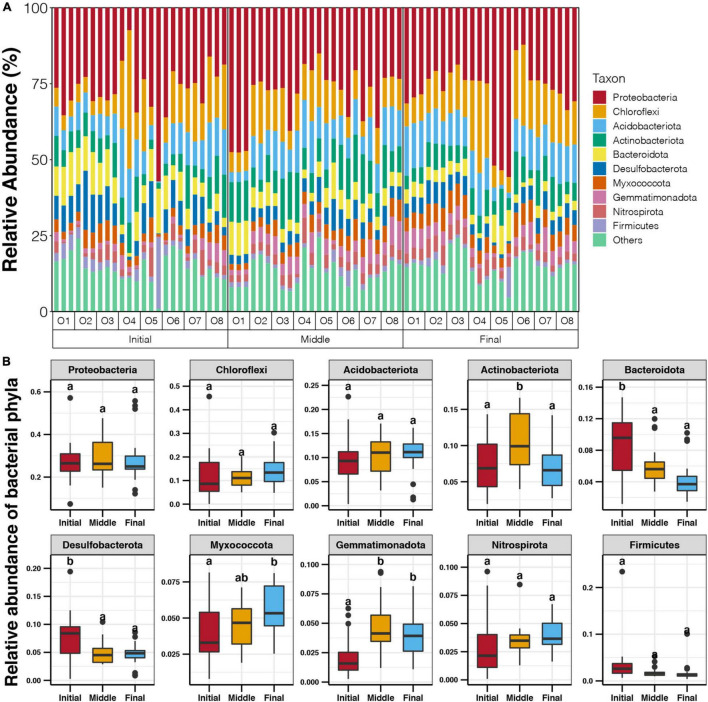
**(A)** Relative abundance of dominant benthic bacterial phyla in the purification area of the IPRS at different locations during the culture cycle. **(B)** Variations in the relative abundance of dominant benthic bacterial phyla in the purification area of the IPRS during the culture cycle. Different lowercases letters above each box in the same subfigure represent significant differences between groups (Tukey’s HSD test, *p* < 0.05).

Proteobacteria (7.4–57.2%) was the most dominant phylum in the sediments of the purification area, followed by Chloroflexi (0.1–45.6%), Acidobacteriota (0.4–22.7%), Actinobacteriota (1.9–16.6%), and Bacteroidota (1.2–14.7%) (Figure 3A). The phyla Desulfobacterota, Myxococcota, Gemmatimonadota, Nitrospirota, and Firmicutes also contributed a considerable proportion of the benthic bacterial communities, with average values of 5.8, 4.7, 3.6, 3.5, and 2.3%, respectively (Figure 3A). As the culture cycle progressed, the relative abundances of Bacteroidota and Desulfobacterota in the sediments of the purification area gradually decreased (Tukey’s HSD test, *p* < 0.05, Figure 3B), whereas those of Myxococcota and Gemmatimonadota increased from the initial stage to the final stage (Tukey’s HSD test, *p* < 0.05, Figure 3B). The relative abundance of Actinobacteriota was significantly higher in sediments in the middle stage compared with those in the initial and final stages (Tukey’s HSD test, *p* < 0.05, Figure 3B).

The collinearity of the bacterial phylum abundances and the sediment characteristics were compared, and a significant correlation was found only for the relative abundance of Gemmatimonadota and the TP content at the middle stage (Spearman correlation, *p* < 0.05, Supplementary Figure 2).

**FIGURE 4:**
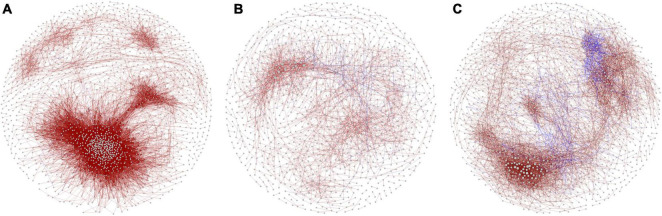
Co-occurrence networks of benthic bacterial communities in the purification area of the IPRS at the **(A)** initial, **(B)** middle, and **(C)** final stages of the culture cycle.

Analysis of the co-occurrence patterns among benthic bacterial taxa in the purification area of the IPRS during the culture cycle showed that the co-occurrence network was markedly more complex at the initial stage compared with the middle and final stages (Figure 4). The co-occurrence networks in the initial, middle, and final stages consisted of 1,638 nodes with 34,860 edges, 1,136 nodes with 2,839 edges, and 1,509 nodes with 7,852 edges, respectively (Table 1). The average degrees of the co-occurrence networks in the initial, middle, and final stages were 42.56, 5.00, and 10.41, respectively (Table 1). In contrast, the average path length and modularity of the networks were 4.84 and 0.297 at the initial stage, 6.44 and 0.739 at the middle stage, and 5.39 and 0.657 at the final stage (Table 1). These results indicate that the network in the benthic bacterial community was most complex at the initial stage, second most complex at the final stage, and simplest at the middle stage. To assess the stability of the benthic bacterial communities, we compared the ratio of positive to negative edges in the networks among the three stages. As the culture cycle progressed, this ratio increased consistently from 0.04% at the initial stage to 6.97% at the middle stage to 7.28% at the final stage (Table 1). These findings suggest that the benthic bacterial community in the purification area of the IPRS tended to become more stable over the course of the culture cycle.

**TABLE 1:** Topological parameters of co-occurrence networks based on benthic bacterial communities in the purification area of the IPRS during the culture cycle.

	Initial	Middle	Final
Nodes	1638	1136	1509
Edges	34860	2839	7852
Degree	42.564	4.998	10.407
Modularity	0.297	0.739	0.657
Average path length	4.84	6.44	5.386
Positive edge ratio	99.96%	93.03%	92.72%
Negative edge ratio	0.04%	6.97%	7.28%

### Mechanisms Involved in Regulating Benthic Bacterial Community Structure

**FIGURE 5:**
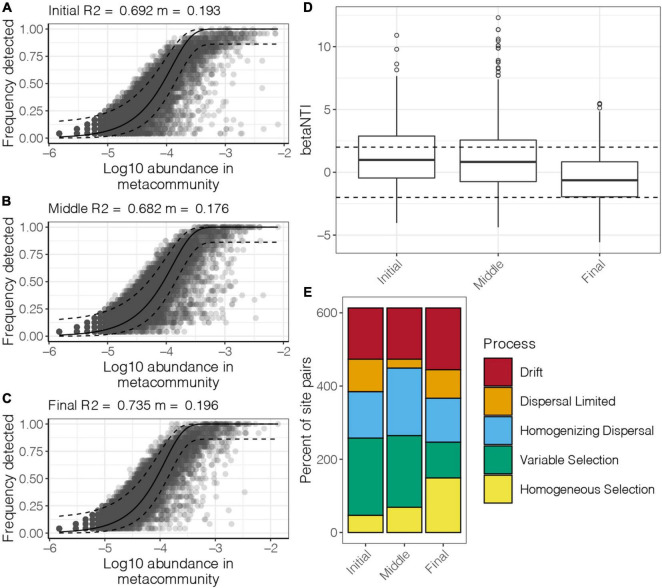
Fit of the NCM for benthic bacterial communities in the purification area of the IPRS at the **(A)** initial, **(B)** middle, and **(C)** final stages of the culture cycle. The solid and dashed lines indicate the best fit to the NCM and 95% confidence intervals around the model prediction, respectively. *m* indicates the meta-community size times immigration, and *R*^2^ indicates the fit to this model. **(D)** Distribution of the βNTI of the benthic bacterial communities in the purification area of the IPRS at different culture stages. **(E)** Comparison of the contribution of the ecological processes that determine community structure of benthic bacterial communities in the purification area of the IPRS at different culture stages.

To explore mechanisms responsible for the observed spatiotemporal patterns, the relative role of niche and neutral processes in community assembly were analyzed. The NCM successfully estimated a large fraction of the relationship between the occurrence frequency of OTUs and their relative abundance variations. About 70% of community variance in the benthic bacterial communities in the purification area at different culture stages were explained by the NCM (Figures 5A–C). These results indicated that stochastic processes were very important in shaping the benthic bacterial communities in the purification area. The *m*-value was estimated to range from 0.176 to 0.196 at different culture stages. A high *m*-value represents high species dispersal ability. The NCM results indicated that species dispersal of benthic bacterial communities was similar in the initial and final stages and was slightly higher than that in the middle stage of the culture cycle.

Null model analysis was carried out to classify benthic bacterial community pairs into underlying driving forces of homogeneous selection, heterogeneous selection, dispersal limitation, homogenizing dispersal, and drift. Overall, stochastic processes (dispersal limitation, homogenizing dispersal, and drift) contributed the most to the structure of benthic bacterial communities in the purification area of the IPRS (the median of βNTI in all culture stages was between –2 and 2, Figure 5D). Of these processes, homogenizing dispersal had a greater effect than dispersal limitation (Figure 5E). Moreover, heterogeneous selection was the dominant deterministic process for structuring the benthic bacterial communities in the initial and middle stages, whereas homogeneous selection contributed more to the final stage of the culture cycle.

## Discussion

### Dynamics of Benthic Bacterial Communities in the Purification Area of the In-Pond Raceway Recirculating Culture System

Significant variations in the diversity of bacterial communities during the culture process have been reported for numerous aquaculture systems (Guan et al., 2020; Zhao et al., 2020). In contrast, we detected no obvious differences in the richness and diversity of benthic bacterial communities among different culture stages in the IRPS. The IPRS used in our study is a completely closed aquaculture system, and there is no water exchange with the external environment during the culture cycle. The fixed species pool and continuous water circulation may explain the observed stability of benthic bacterial diversity in the purification area of the IPRS.

In contrast to the stable alpha diversity indices, the composition of benthic bacterial communities in the purification area of the IPRS demonstrated substantial variation during the culture cycle (Figure 2). This result indicated that the abundances of major bacterial taxa in the IPRS sediment changed during the culture cycle. Bacteroidota and Desulfobacterota were more abundant in the sediments of the purification area at the initial stage compared to other culture stages (Figure 3B). Bacteroidota are increasingly regarded as specialists involved in the degradation of high molecular weight organic matter (proteins and carbohydrates) (Li et al., 2017). The sequencing of Bacteroidota genomes has confirmed the presence of numerous carbohydrate-active enzymes covering a large spectrum of substrates from plant, algal, and animal origin (Han et al., 2009; Qin et al., 2010). Desulfobacterota can transport electrons from hydrogen sulfide to nitrate or nitrite (Risgaard-Petersen et al., 2014), and some Desulfobulbaceae have been shown to reduce sulfates into sulfides (Jiang et al., 2010). These abilities are very important for degrading organic waste such as food and feces, improving resource utilization efficiency, and maintaining the stability of the entire IPRS. The decrease in the abundance of these two groups over time suggests that organic matter degradation and carbon and sulfur cycling mediated by the benthic bacterial communities could inhibit in the purification area of the IPRS over the course of the culture cycle.

For a deeper investigation of the distribution patterns of benthic bacteria in the purification area of the IPRS, we constructed co-occurrence networks. The bacterial community at the initial stage of the culture cycle was more complex, with remarkably higher numbers of nodes and edges, compared with the other time points (Figure 4). High connectivity means that community members share more similar ecological characteristics and express as a high degree of functional redundancy (Yao et al., 2014). This was not unexpected because the bacterial communities in the purification area of the IPRS originated from the natural environment and had not been subjected to stressors related to aquaculture activities. The co-occurrence networks also revealed that most interspecies correlations were positive for all significant correlations (Table 1). These results somewhat reflected cross-feeding, co-colonization, and co-evolution among different species (Faust and Raes, 2012), which could be due to the closeness and connectivity in the IPRS. More importantly, the ratio of negative to positive correlations in the co-occurrence networks increased as the culture cycle progressed (Table 1). This higher ratio in the ecological networks indicated more stable bacterial communities in the ecosystem (Coyte et al., 2015). Taken together, our results indicate that the abundance of some microorganisms in the benthic microbial community in the purification area of the IPRS gradually decreased with the progression of the culture cycle due to the functions of these microorganisms were alternative by other members. Thus, as the reduced redundancy of the benthic bacterial communities during the progression of the culture cycle, a less complex but more stable community structure developed by the end of the cycle.

### Relationships Between Bacterial Communities and Environmental Factors in the Purification Area of the In-Pond Raceway Recirculating Culture System

We detected significant variations in both sediment characteristics and the diversity and composition of benthic bacterial communities in the studied IPRS among the three culture stages (Figures 1**–**3). However, we found a very limited correlation between the sediment characteristics and the benthic bacterial communities (Supplementary Tables 1–5 and Supplementary Figure 2). Changes in environmental conditions leading to changes in bacterial community compositions have been reported frequently in many environments (Zhao et al., 2018; Rath et al., 2019; Ouyang et al., 2020). Hou et al. (2017) analyzed relationships between the bacterial communities in the water and environmental factors in shrimp culture enclosure ecosystems and found that TP and TN were the most important factors shaping bacterial community structure. In another study, large-scale cultivation of seaweed decreased the inorganic nutrient contents, which significantly altered the water bacterial community compositions in the cultivation zone (Zhao et al., 2018). Moreover, Auffret et al. (2013) found that higher water quality resulted in a more stable bacterial community with a higher nitrification ability and reduced geosmin production in RASs. Most of these studied focused on the bacterial communities in the water. In contrast, few studies have evaluated the effects of physical and chemical factors related to benthic bacterial communities in the sediments of RASs (Guan et al., 2020). The composition of sediment is very complex, and the physicochemical characteristics of sediment can be extremely distinct even within a very small area (Avramidis et al., 2013). Thus, the results from a specific area or ecosystem are not universally applicable.

The limited correlation between the sediment characteristics and the benthic bacterial communities observed in the present study could be due to the organic pollutants have been effectively removed in the initial and middle stages of the culture activities. The concentrations of TN, TP, and TOC in the sediments were all significantly lower in the final stage compared to those in the initial and middle stages (Figure 1A). The lowest concentration of these organic matters in the final stage could result in the lower abundances of bacteria with the ability to use them, such as Bacteroidota and Desulfobacterota (Figure 3B). This could be the reason of the weaken abilities of organic matter degradation and carbon and sulfur cycling in the benthic bacterial communities at the final stage. Moreover, the decreased abundances of these microorganisms in the final stage also contributed to the formation of a more stable bacterial community because the need for reduced redundancy.

### Stochastic Processes Dominate the Community Structure of Benthic Bacteria in the Purification Area of the In-Pond Raceway Recirculating Culture System

Understanding the contribution of ecological processes to the structure of bacterial communities is a crucial issue in microbial ecology (Zhou and Ning, 2017). In this study, we used two different models based on the neutral and niche concepts to analyze benthic bacterial community structure in the purification area of the IPRS during the culture cycle. These models are useful for assessing the assembly mechanisms of bacterial communities in diverse environments. Our results revealed that stochastic processes overwhelmed deterministic processes in determining the IPRS benthic bacterial community structure (Figure 5). This finding is in agreement with the limited correlations between the sediment characteristics and benthic bacterial communities observed in this study. Similarly, results of a field experiment conducted in the Inner Mongolian grasslands indicated that bacterial community structure was not environmentally determined but instead was governed by stochastic processes (Hao et al., 2016). A global-scale study of bacterial communities in 269 wastewater treatment plants in 23 countries on 6 continents indicated that the spatial turnover of bacterial communities was largely driven by stochastic processes (Wu et al., 2019). We also found that homogenizing dispersal was the strongest stochastic process shaping the benthic bacterial community structure (Figure 5E). During the culture cycle, continuous water circulation kept the whole IPRS in a relatively consistent state and effectively promoted the dispersal of bacteria. This powerful hydrologic mixing can increase dispersal-related processes and ecological drift, making stochastic processes more prominent (Meyerhof et al., 2016).

## Conclusion

In conclusion, the aquaculture waste deposited from the tail water during recirculation significantly affected sediment characteristics in the purification area at the different culture stages. However, these significant differences in sediment characteristics had limited impact on the benthic bacterial communities. Stochastic processes dominated the structure and dynamics of benthic bacterial communities in the purification area of the IPRS, especially homogenizing dispersal. Homogenizing dispersal was particularly enhanced by powerful hydrologic mixing caused by continuous water circulation. By the end of the culture cycle, a less complex but more stable benthic bacterial community had formed in the purification area, and it was characterized by potentially inhibited organic matter degradation and carbon and sulfur cycling abilities. Therefore, as organic waste continued to settle, the benthic microbial communities with weaken abilities of organic matter degradation and carbon and sulfur cycling in the purification area were not very well matched to the intended purification function of the purification area. Although a more uniform and stable benthic microbial community was uncovered, we should improve the IPRS, especially the purification area by somewhat methods to better reduce environmental pollution.

## Data Availability Statement

The datasets presented in this study can be found in online repositories. The names of the repository/repositories and accession number(s) can be found below: https://www.ncbi.nlm.nih.gov/bioproject.

## Author Contributions

YH: conceptualization, methodology, formal analysis, data curation, and writing-original draft preparation. BL: visualization and investigation. GX: visualization and writing- reviewing and editing. DL and RJ: writing- reviewing and editing. CZ: investigation. JZ: resources, writing- reviewing and editing, and supervision. All authors contributed to the article and approved the submitted version.

## Conflict of Interest

The authors declare that the research was conducted in the absence of any commercial or financial relationships that could be construed as a potential conflict of interest.

## Publisher’s Note

All claims expressed in this article are solely those of the authors and do not necessarily represent those of their affiliated organizations, or those of the publisher, the editors and the reviewers. Any product that may be evaluated in this article, or claim that may be made by its manufacturer, is not guaranteed or endorsed by the publisher.
