# Supplementary material for: Dynamic and Assembly of Benthic Bacterial Community in an Industrial-Scale In-Pond Raceway Recirculating Culture System
Source: Front Microbiol. 2021 Dec 23;12:797817. doi: 10.3389/fmicb.2021.797817 (PMC8733461; doi:10.3389/fmicb.2021.797817)
Supplement: Supplementary file 1 [file Table_1.DOCX]

### Supporting Information for

### Stochastic processes dominate the benthic bacterial community assembly in an industrial-scale in-pond raceway recirculating culture system

Yiran Hou^1, 2^ , Bing Li^1, 2, *^, Gangchun Xu^1, 2^, Da Li^3^, Chengfeng Zhang^1, 2^, Rui Jia^1, 2^, Quanjie Li^1^, Jian Zhu^1, 2, *^

1 Key Laboratory of Freshwater Fisheries and Germplasm Resources Utilization, Ministry of Agriculture and Rural Affairs, Freshwater Fisheries Research Center, Chinese Academy of Fishery Sciences, Wuxi 214081, China

2 Wuxi Fisheries College, Nanjing Agricultural University, Wuxi 214081, China

3 Ocean and Fishery Research Institute of Rizhao, Rizhao 276800, China

* Corresponding author

E-mail: [zhuj@ffrc.cn](mailto:zhuj@ffrc.cn)

Tel.: +86-510-85550535

Fax: +86-510-85551464


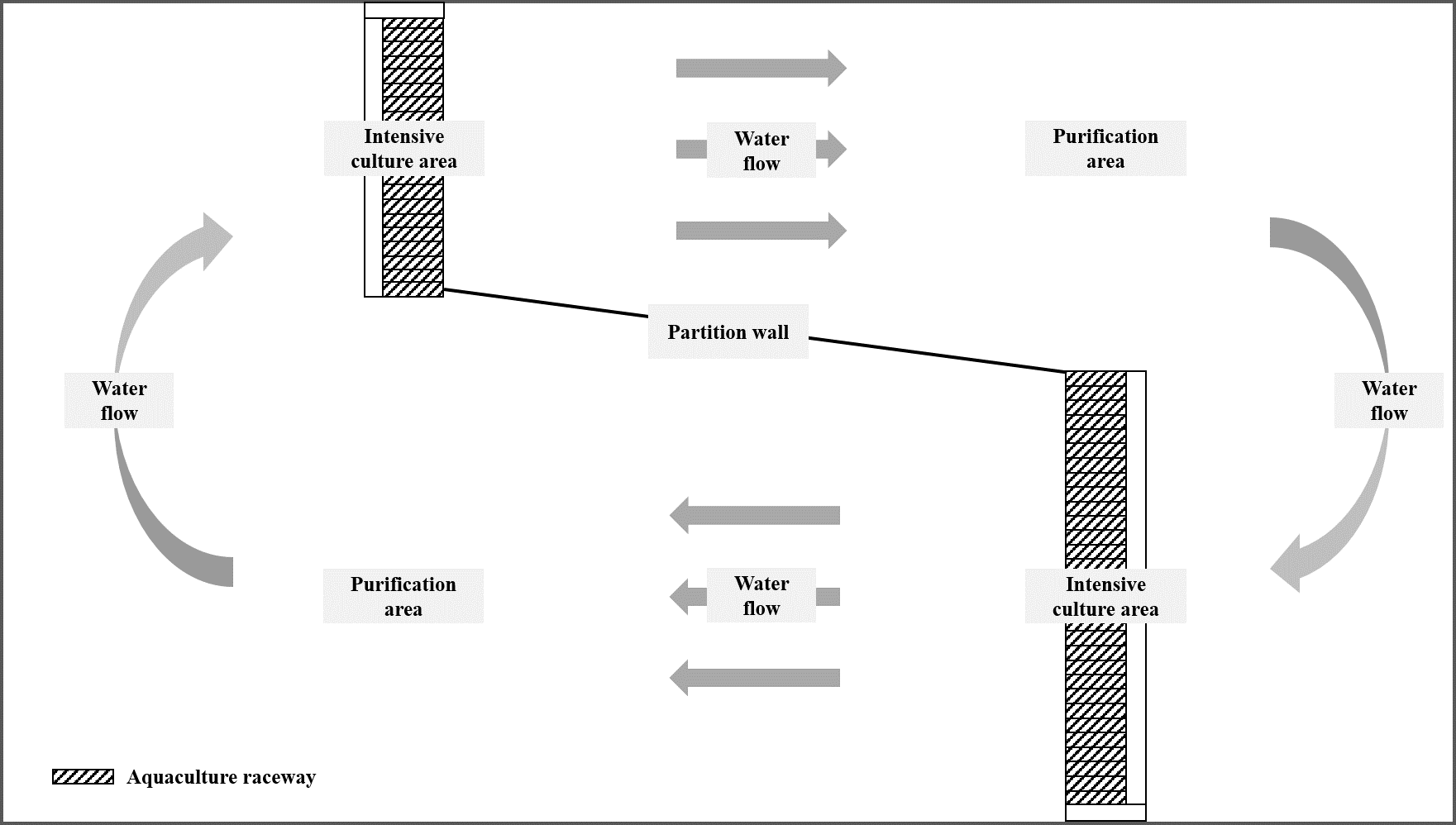


**Figure S1.** Schematic diagram for the studied in‐pond raceway recirculating culture system (IPRS).


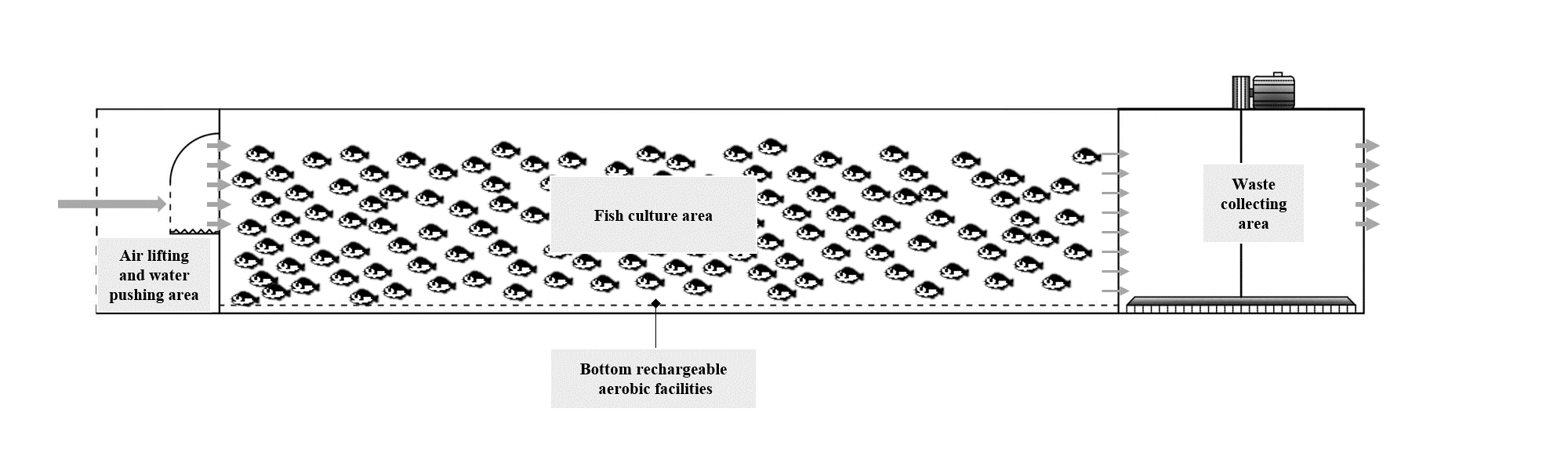


**Figure S2.** Schematic diagram for the aquaculture raceway in the in‐pond raceway recirculating culture system (IPRS). Arrow indicates the direction of water flow.

**Figure S3.** Correlations between the relative abundance of bacterial phyla and the sediment characteristics in the purification area of the in‐pond raceway recirculating culture system (IPRS) at the middle stage. “+” represents the p-value of Spearman’s correlation lower than 0.05.

**Table S1.** Sediment characteristics in the purification area of the in‐pond raceway recirculating culture system (IPRS) during the full culture-cycle.

| **Sediment characteristics** | Initial | Middle | Final |
| --- | --- | --- | --- |
| **TN** | 1.746±1.494^a^ | 3.131±1.407^b^ | 3.022±0.977^b^ |
| **TP** | 0.343±0.127^a^ | 0.281±0.090^ab^ | 0.240±0.085^a^ |
| **TOC** | 0.063±0.011^a^ | 0.079±0.032^b^ | 0.054±0.011^a^ |

**Note.** Data are expressed as mean values ± SEM (n = 24). Means in the same row with different superscripts are signiﬁcantly different (Tukey’s HSD test, *p* < 0.05).

**Table S2.** Sediment characteristics in the purification area of the in‐pond raceway recirculating culture system (IPRS) from different locations during the full culture-cycle.

| Location | Initial | | | Middle | | | Final | | |
| --- | --- | --- | --- | --- | --- | --- | --- | --- | --- |
|  | TN | TP | TOC | TN | TP | TOC | TN | TP | TOC |
| O1 | 2.464±2.542^a^ | 0.412±0.115^a^ | 0.066±0.014^ab^ | 2.782±1.556^c^ | 0.219±0.014^c^ | 0.075±0.029^a^ | 3.257±1.040^a^ | 0.259±0.031^a^ | 0.053±0.002^a^ |
| O2 | 1.458±1.057^a^ | 0.439±0.070^a^ | 0.058±0.003^ab^ | 2.794±0.232^ab^ | 0.279±0.044^bc^ | 0.105±0.048^a^ | 3.027±0.983^a^ | 0.257±0.022^a^ | 0.052±0.002^a^ |
| O3 | 1.210±0.668^a^ | 0.465±0.211^a^ | 0.064±0.014^ab^ | 3.964±0.421^a^ | 0.289±0.015^a^ | 0.063±0.018^a^ | 1.988±1.109^a^ | 0.163±0.021^a^ | 0.058±0.004^a^ |
| O4 | 3.135±1.968^a^ | 0.280±0.046^a^ | 0.074±0.009^a^ | 4.570±0.998^bc^ | 0.340±0.052^ab^ | 0.105±0.033^a^ | 3.176±0.733^a^ | 0.186±0.026^a^ | 0.047±0.028^a^ |
| O5 | 0.449±0.193^a^ | 0.245±0.107^a^ | 0.047±0.001^b^ | 3.729±1.391^c^ | 0.229±0.018^bc^ | 0.060±0.012^a^ | 2.367±0.352^a^ | 0.309±0.043^a^ | 0.042±0.003^a^ |
| O6 | 2.690±0.273^a^ | 0.315±0.125^a^ | 0.073±0.010^ab^ | 1.043±0.435^bc^ | 0.154±0.075^b^ | 0.111±0.020^a^ | 2.968±0.794^a^ | 0.165±0.076^a^ | 0.063±0.007^a^ |
| O7 | 1.226±2.039^a^ | 0.230±0.023^a^ | 0.065±0.011^ab^ | 1.767±0.081^ac^ | 0.306±0.081^ab^ | 0.051±0.002^a^ | 3.072±1.148^a^ | 0.252±0.137^a^ | 0.057±0.004^a^ |
| O8 | 1.323±0.869^a^ | 0.359±0.095^a^ | 0.059±0.005^ab^ | 4.398±0.463^ac^ | 0.433±0.026^ab^ | 0.059±0.004^a^ | 4.319±0.437^a^ | 0.332±0.110^a^ | 0.059±0.002^a^ |

**Note.** Data are expressed as mean values ± SEM (n = 3). Means in the same column with different superscripts are signiﬁcantly different (Tukey’s HSD test, *p* < 0.05).

**Table S3.** Alpha diversity indices of benthic bacterial communities in the purification area of the in‐pond raceway recirculating culture system (IPRS) during the full culture-cycle.

| **Alpha diversity** | Initial | Middle | Final |
| --- | --- | --- | --- |
| **Chao1** | 7980±2004^a^ | 8146±1559^a^ | 8195±2370^a^ |
| **Shannon** | 7.238±0.822^a^ | 7.396±0.424^a^ | 7.232±0.504^a^ |

**Note.** Data are expressed as mean values ± SEM (n = 24). Means in the same row with different superscripts are signiﬁcantly different (Tukey’s HSD test, *p* < 0.05).

**Table S4.** Sediment characteristics in the purification area of the in‐pond raceway recirculating culture system (IPRS) from different locations during the full culture-cycle.

| Location | Initial | | | Middle | | Final | | |
| --- | --- | --- | --- | --- | --- | --- | --- | --- |
|  | Chao1 | Shannon | Chao1 | | Shannon | | Chao1 | Shannon |
| O1 | 8617±650^a^ | 7.523±0.053^a^ | 8211±926^ac^ | | 7.514±0.238^ac^ | | 6817±862^ac^ | 6.860±0.188^ab^ |
| O2 | 8859±339^a^ | 7.520±0.234^a^ | 8019±563^ac^ | | 7.453±0.259^ac^ | | 8568±1208^bcd^ | 7.470±0.270^bc^ |
| O3 | 8304±743^a^ | 7.447±0.203^a^ | 9823±582^ac^ | | 7.763±0.068^bc^ | | 6252±516^ab^ | 6.935±0.122^ab^ |
| O4 | 6321±4392^a^ | 6.317±2.162^a^ | 7828±626^ac^ | | 7.549±0.195^bc^ | | 4925±913^a^ | 6.429±0.396^a^ |
| O5 | 5083±885^a^ | 6.527±0.359^a^ | 6447±708^a^ | | 7.098±0.076^ab^ | | 7558±1119^acd^ | 7.146±0.402^ac^ |
| O6 | 9428±805^a^ | 7.692±0.125^a^ | 6896±2057^a^ | | 7.078±0.602^ab^ | | 9397±1884^ce^ | 7.477±0.293^bc^ |
| O7 | 9123±489^a^ | 7.478±0.255^a^ | 10449±1269^c^ | | 7.928±0.093^c^ | | 9953±736^de^ | 7.685±0.075^c^ |
| O8 | 8106±611^a^ | 7.396±0.157^a^ | 7495±152^ab^ | | 6.788±0.005^a^ | | 12088±1026e | 7.852±0.113^c^ |

**Note.** Data are expressed as mean values ± SEM (n = 3). Means in the same column with different superscripts are signiﬁcantly different (Tukey’s HSD test, *p* < 0.05).

**Table S5.** Mantel tests of benthic bacterial communities and sediment characteristics in the purification area of the in‐pond raceway recirculating culture system (IPRS) during the full culture-cycle.

| **Sediment characteristics** | All | | Initial | | Middle | | Final | |
| --- | --- | --- | --- | --- | --- | --- | --- | --- |
|  | r | p | r | p | r | p | r | p |
| **TN** | 0.076 | 0.061 | 0.131 | 0.135 | 0.015 | 0.405 | -0.018 | 0.544 |
| **TP** | -0.025 | 0.639 | 0.001 | 0.410 | -0.088 | 0.788 | 0.089 | 0.189 |
| **TOC** | 0.027 | 0.332 | 0.036 | 0.340 | -0.066 | 0.739 | 0.312 | 0.039 |
